# Supplementary material for: Hierarchical Surface Structures and Large-Area Nanoscale Gratings in As2S3 and As2Se3 Films Irradiated with Femtosecond Laser Pulses
Source: Materials (Basel). 2023 Jun 22;16(13):4524. doi: 10.3390/ma16134524 (PMC10342740; doi:10.3390/ma16134524)
Supplement: Supplementary file 1 [file materials-16-04524-s001.zip › materials-2440072-supplementary.pdf]

EDX analysis of the initial and irradiated As_2S_3 and As_2Se_3 films

To accurately establish the stoichiometric ratios of the As_2S_3 and As_2Se_3 films material and determine its uniformity, the energy-dispersive X-ray spectroscopy (EDX) analysis of structures was carried out. The irradiated As_2S_3 and As_2Se_3 surfaces were also analyzed, and results were compared to initial films.

The chemical composition of both the initial and irradiated As_2S_3 and As_2Se_3 films was evaluated using scanning electron microscope (SEM) JEOL JSM-6010 (JEOL Ltd., Japan) equipped with an energy-dispersive spectrometer JEOL EX94400T4L11.

Additionally chemical composition, including the profile distributions of the chemical elements across the irradiated As_2Se_3 film thickness were investigated by transmission electron microscope (TEM) (JEOL JEM 2100 Plus (JEOL Ltd., Japan) equipped with a JEOL EX-24261M1G5T energy dispersive analyzer. TEM cross-section specimens were prepared using dual-beam workstation Helios G4CX FEI with micromanipulator by the in-situ lift-out method. The As_2Se_3 sample irradiated by femtosecond laser pulses in scanning mode (fluence $F = 50$ mJ/cm^2, number of pulses per unit area $N = 800$), was used.

The EDX analysis demonstrated that the stoichiometry of both the initial and irradiated As_2Se_3 films correspond to 40±1% of As and 60±1% of Se, and there is no change in the composition of the substance after the film irradiation (Figure 1). The same result is obtained for As_2S_3 films: the stoichiometry of initial film and irradiated area correspond to 40±1% of As and 60±1% of S. The maps demonstrate uniform distribution of the chemical elements across the film surface.

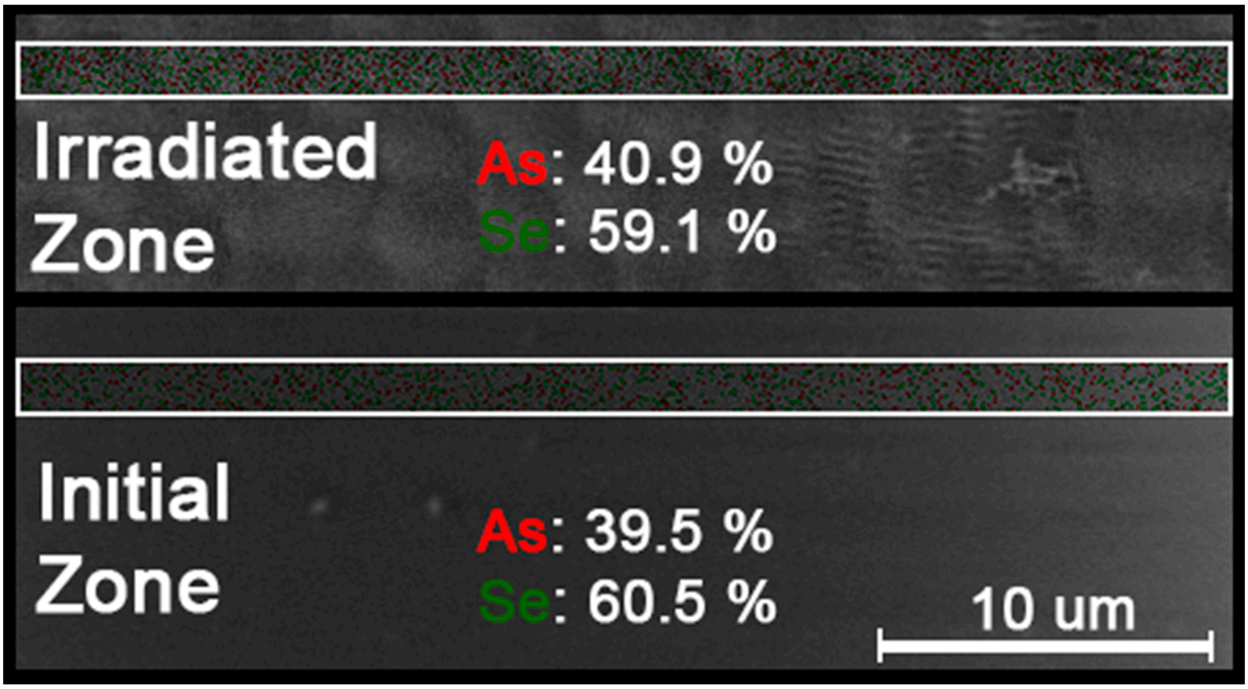

Figure S1. EDX analysis maps for initial (lower panel) and irradiated (upper panel) zones of As_2Se_3 films.

The EDX results obtained for the irradiated As_2Se_3 film in TEM also demonstrate the composition corresponding to 40±10% of As and 60±10% of Se as well as uniform distribution of the chemical elements across the film thickness (Figure 2).

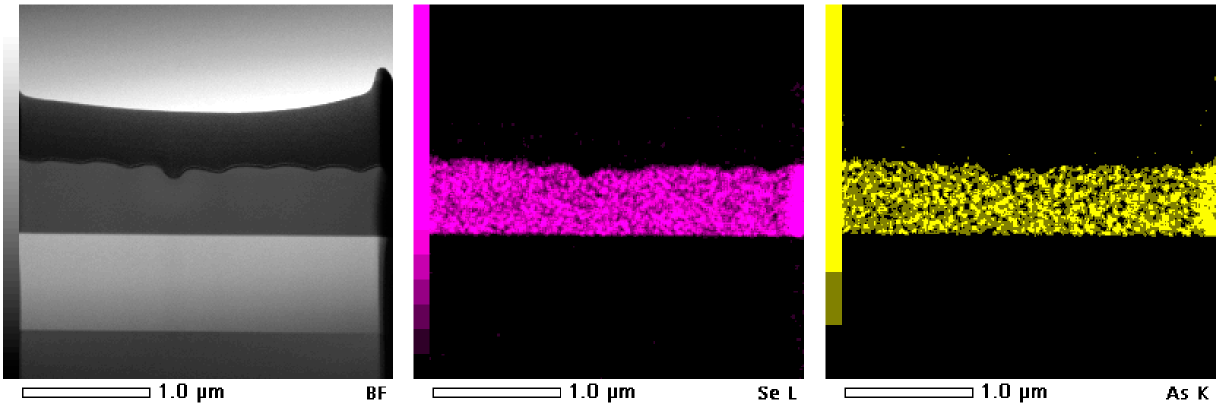

Figure S2. TEM image of the As_2Se_3 cross-section (left panel), EDX analysis results for the cross-section of the irradiated As_2Se_3 film: Se (central panel) and As (right panel) distribution.
